# A patient with an adrenal metastasis and periadrenal lymph node metastases of a laryngeal squamous cell carcinoma: a case report

**DOI:** 10.1186/s13256-025-05731-z

**Published:** 2025-12-22

**Authors:** Oliver Gimm, Christer M. Nilsson, Aristotelis Stefanis

**Affiliations:** 1 Department of Biomedical and Clinical Sciences, Linköping University, Linköping, Sweden; 2 Clinical Department of Surgery in Linköping, Region Östergötland, Linköping, Sweden; 3 Clinical Department of Pathology in Linköping, Region Östergötland, Linköping, Sweden; 4 Clinical Department of Oncology in Linköping, Region Östergötland, Linköping, Sweden

**Keywords:** Laryngeal squamous cell carcinoma, Adrenal metastasis, Immunotherapy, PD-1 inhibitors, Case report

## Abstract

**Background:**

Adrenal tumors are quite common. If malignancy is suspected, the question arises whether the adrenal tumor is the primary or a secondary (= metastasis) malignancy. Surgery on adrenal metastases is generally considered to be of limited value if other distant metastases exist. We present a case of laryngeal squamous cell cancer that subsequently developed a large adrenal metastasis and multiple lymph node metastases treated successfully with surgery and immunotherapy.

**Case presentation:**

The white male patient in his 60s was treated 20 months earlier with radiochemotherapy owing to laryngeal squamous cell cancer and considered to be in complete remission. He was now under investigation owing to macroscopic hematuria. Computed tomography showed an unrelated large left adrenal mass and periadrenal enlarged lymph nodes. A primary adrenal neoplasm with locoregional lymph node metastases was suspected. The patient underwent open adrenalectomy including removal of the locoregional lymph nodes. Histology surprisingly revealed that the large adrenal tumor was a metastasis of the laryngeal squamous cell cancer. In addition, three out of five lymph nodes removed also contained metastases of the same type of squamous cell cancer. Additional lymph node metastases (para-aortic, mediastinum, and right axilla) were subsequently diagnosed, and the patient was treated with immunotherapy. About 6 months later, the patient was in complete remission. Immunotherapy was continued and eventually discontinued after the completion of a 2-year treatment. A total of 3 years after the adrenal surgery, the patient is still considered to be in complete remission.

**Conclusion:**

Despite the overall bad prognosis of metastasized laryngeal squamous cell carcinoma, multidisciplinary and multimodal treatment can lead to complete remission for several years.

## Background

Adrenal tumors are quite common. They are looked for when adrenal hormone overproduction has been suspected and diagnosed. More often, however, they are found incidentally when performing abdominal imaging for other reasons. The prevalence of incidentally detected adrenal lesions in patients with or without malignant disease undergoing computed tomography (CT) is about 4–5% [1]. In these instances, determining the dignity of the adrenal tumor can become a diagnostic challenge. To help making the decision which patient should undergo surgery and which one may not, mainly radiologic properties and size are used. With regard to the former, if the Hounsfield unit (HU) of a homogenous adrenal lesion on an unenhanced CT scan is < 10, the lesion is considered benign [2, 3]. With regard to the latter, adrenal lesions > 4 cm are generally recommended to be removed surgically unless a myelolipoma is diagnosed [2, 3]. Biopsy of the adrenal tumor is generally only reserved for patients in whom the results would change the overall disease management [3]. In case the adrenal tumor is a suspected to be an adrenocortical cancer (ACC), biopsy is generally not recommended because of needle tract metastases and the limited diagnostic value in differentiating benign from malignant lesions [4]. In patients with pheochromocytoma, biopsy may lead to severe complications [5].

In any case, when malignancy is suspected, the question arises whether the adrenal tumor is the primary or a secondary (= metastasis) malignancy. Primary malignant tumors of the adrenal gland include ACC and malignant pheochromocytomas [2, 3]. If technically feasible, surgery is generally recommended. Regarding secondary malignancy, probably all tumors can metastasize to the adrenal gland. Tumors that are known to metastasize relatively often to the adrenal gland include lung cancer, kidney cancer, breast cancer, malignant melanoma, and colon cancer [3] with lung cancer being the most common primary malignancy [6, 7]. In rare instances, the origin of the primary tumor that has metastasized to the adrenal gland cannot be determined (carcinoma of unknown primary [CUP]) [8]. Surgery on adrenal metastases is generally considered to be of limited value if other distant metastases exist [9].

We present a case of laryngeal squamous cell cancer that subsequently developed a large adrenal metastasis and multiple lymph node metastases treated with surgery and immunotherapy leading to several years of complete remission.

## Case presentation

**Fig. 1:**
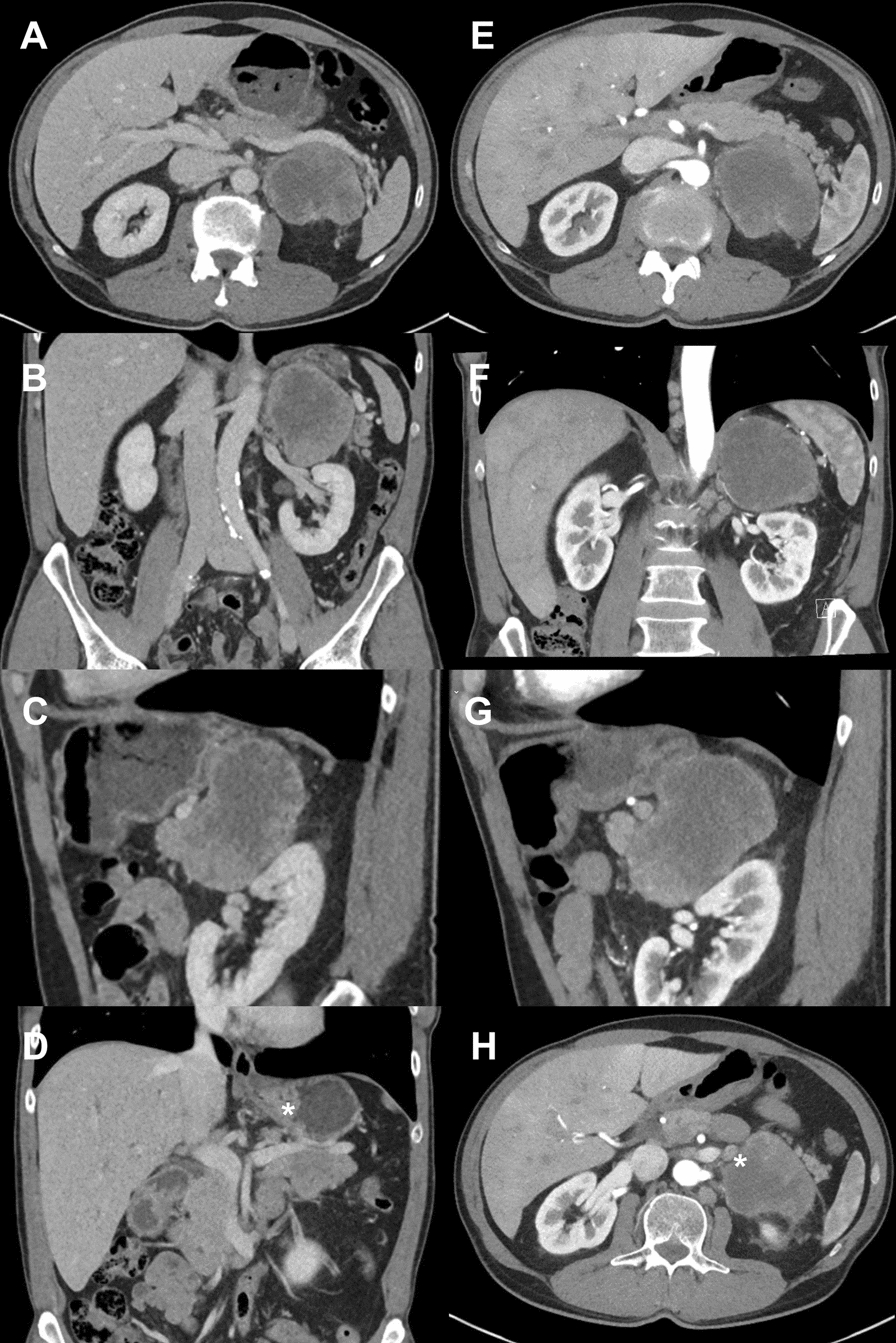
Computed tomography. **A–D** First computed tomography of the abdomen showing the large adrenal tumor on the left side; transverse (**A**), coronal (**B**), sagittal (**C**) section. ^*^Thickened gastric wall suspected to indicate infiltration of the adrenal tumor into the stomach (**D**). **E**–**H** Follow-up computed tomography a few weeks after the first computed tomography of the abdomen showing an increase in the large adrenal tumor on the left side; transverse (**E**), coronal (**F**), sagittal (**G**) section. ^*^Enlarged lymph node (14 mm) adjacent to the large adrenal tumor suspected to indicate a lymph node metastasis of the adrenal tumor (**H**)

We present a white male patient in his 60s who was referred to the Department of Surgery by the Department of Urology, University Hospital, Linköping, Sweden, owing to a large tumor in the left adrenal gland (Fig. 1A–C). The tumor was identified during the workup of macroscopic hematuria that was considered to be unrelated to the large adrenal tumor (maximum diameter 80 mm). Owing to suspected infiltration of the stomach (Fig. 1D), the patient underwent gastroscopy that did not show any signs of infiltration of the large adrenal mass.

The patient had a previous history of squamous cell cancer of the larynx (pT3 cN1 M0) that was diagnosed and treated with a transient tracheostomy, external radiation (68 Gy in total), and concomitant chemotherapy (cisplatin) about 20 months earlier. He had since been in remission with no signs of recurrence or metastases.

### Investigations

For better planning of the operation, the patient underwent thoracoabdominal CT scan a few weeks after the first CT that showed progress of the adrenal mass; the largest diameter was now 90 mm (Fig. 1E–G). In addition, enlarged locoregional lymph nodes up to 14 mm were noted (Fig. 1H).

The biochemical analyses showed no hormone overproduction. The steroid hormone profile was also not pathologic.

### Treatment

A primary adrenal neoplasm with locoregional lymph node metastases was suspected, and the patient underwent open left-sided adrenalectomy including removal of the locoregional lymph nodes. The postoperative period was uneventful, apart from discomfort in the epigastric region that was later diagnosed as gastritis caused by *Helicobacter pylori* and treated accordingly.

**Fig. 2:**
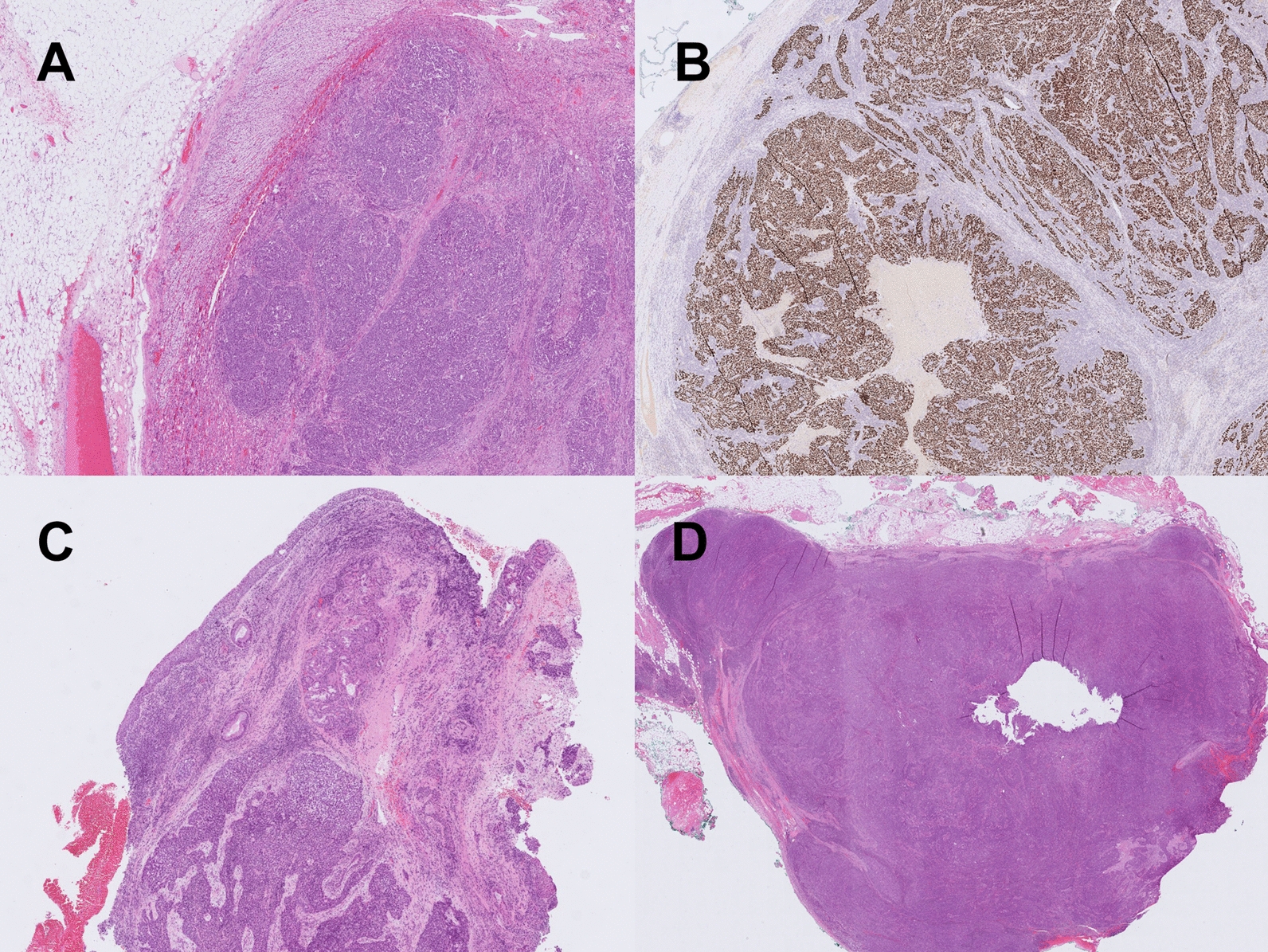
Histology. **A** Hematoxylin and eosin (H&E) staining of resected left adrenal gland showing metastasis of squamous cell carcinoma. **B** Positive p40 staining of the tumor in the left adrenal gland in keeping with metastasis of squamous cell carcinoma. **C** H&E staining of a biopsy from the larynx showing squamous cell carcinoma in primary site. **D** H&E staining of a lymph node adjacent to the left adrenal gland showing metastasis of squamous cell carcinoma

Histology surprisingly revealed that the large tumor in the left adrenal was a metastasis (Fig. 2A, B) of the laryngeal squamous cell cancer that the patient was treated for before (Fig. 2C). In addition, three out of five lymph nodes removed also contained a metastasis of the same squamous cell cancer (Fig. 2D). One lymph node metastasis was found in the renal hilus, and two metastases were diagnosed in para-aortic lymph nodes.

### Outcome and follow-up

During follow-up, additional para-aortic lymph node metastases as well as lymph node metastases in the mediastinum and right axilla were diagnosed. The patient started treatment with PD-1 inhibitor monotherapy which was very well tolerated. About 6 months after the adrenalectomy, the patient was considered to be in complete remission. The treatment with PD-1 inhibitors was discontinued after the completion of a 2-year treatment, and the patient has been actively monitored since then. More than 4.5 years after the initial diagnosis of laryngeal squamous cell carcinoma and 3 years after the adrenalectomy, the patient still is considered to be in complete remission.

## Discussion and conclusion

Squamous cell carcinoma of the larynx most often metastasizes to the lungs, bones, regional lymph nodes, and the liver [10]. Of note, squamous cell carcinoma of the larynx has even been shown to be able to metastasize to organs that otherwise rarely are the targets of metastases, such as the heart [11] or muscles [12]. There are very view reports of laryngeal squamous cell carcinoma that metastasized to the adrenal gland [13]. In the report by Dzialach et al., the adrenal metastasis was considered to be unresectable. In our case, resection could be carried out successfully. Of note, there is one publication of an adenocarcinoma of the larynx that metastasized to the adrenal gland in a cat [14].

Radiochemotherapy (for example, 70 Gy in 35 fractions and three scheduled doses of cisplatin) is a valid treatment option in patients with locally advanced squamous cell carcinoma of the larynx [15]. The 5-year age-standardized relative survival of patients with larynx cancer is about 60% [16]. In contrast, the 1- and 2-year overall survival of patients with metastasized squamous cell carcinoma of the larynx are only about 25–30% and 10–15%, respectively, that is, rather poor [10].

The risk of developing isolated distant metastases in patients with neck squamous cell carcinoma without systemic treatment is about 10% [17]. Low tumor grade and the presence of regional lymph node metastases have been shown to be independent risk factors of isolated distant metastases [17]. Of note, the development of distant metastases has been shown to be more likely (odds ratio [OR] = 5.65) in patients with occult lymph node metastases of laryngeal squamous cell carcinoma [18]. Our patient had clinically suspected lymph node metastases and, thus, an increased risk of developing distant metastases.

It was remarkable that the locoregional lymph nodes of the adrenal gland also contained metastases of the laryngeal squamous cell cancer. Initially, we were wondering whether the adrenal metastasis could have been the source for the locoregional lymph node metastases. Of course, hematogenous spreading would have been possible, but the close relation to the adrenal gland made lymphogenous spreading of malignant cells from the adrenal gland to the regional lymph nodes not unlikely.

However, it has been debated whether metastases can be the origin metastases or not [19, 20]. It also has been discussed whether metastases of metastases would be clinically relevant or not [21]. In a computer model analyzing hepatocellular carcinoma, it was concluded that metastases seeded from metastases are clinically irrelevant and that only the first metastases seeded from the primary tumor contribute significantly to the tumor burden and thus cause patient death [21]. In our patient, additional lymph node metastases (para-aortic, mediastinum, and right axilla) were diagnosed soon after the adrenalectomy. Because of these findings, we now consider hematogenous spreading to the peri-adrenal lymph nodes more likely, but the true origin cannot be determined.

This case is an example that patients with metastasized laryngeal squamous cell carcinoma can be in complete remission for several years when multidisciplinary and multimodal treatment is offered.

## Data Availability

The data that support the conclusions of this article are included within the article.
